# Case Report: Hypergranular Platelets in Vaccine-Induced Thrombotic Thrombocytopenia After ChAdOx1 nCov-19 Vaccination

**DOI:** 10.3389/fcvm.2022.824601

**Published:** 2022-02-09

**Authors:** Shane P. Comer, Ana Le Chevillier, Paulina B. Szklanna, Sarah Kelliher, Khalid Saeed, Steven Cullen, Osasere Edebiri, Tiina O'Neill, Niamh Stephens, Luisa Weiss, Claire A. Murphy, Saraswathi Rajakumar, Alexandra Tierney, Conor Hughes, Áine Lennon, Niamh Moran, Patricia B. Maguire, Fionnuala Ní Áinle, Barry Kevane

**Affiliations:** ^1^Conway SPHERE Research Group, Conway Institute, University College Dublin, Dublin, Ireland; ^2^School of Biomolecular and Biomedical Science, University College Dublin, Dublin, Ireland; ^3^Department of Haematology, Mater Misericordiae University Hospital, Dublin, Ireland; ^4^School of Pharmacy and Biomolecular Sciences, Royal College of Surgeons in Ireland, Dublin, Ireland; ^5^Tallaght University Hospital, Dublin, Ireland; ^6^Conway Institute of Biomolecular and Biomedical Research, University College Dublin, Dublin, Ireland; ^7^Department of Paediatrics, Royal College of Surgeons in Ireland, Dublin, Ireland; ^8^School of Medicine, University College Dublin, Dublin, Ireland; ^9^UCD Institute for Discovery, University College Dublin, Dublin, Ireland; ^10^Department of Haematology, Rotunda Hospital, Dublin, Ireland

**Keywords:** platelets, thrombosis, thrombocytopenia, ChAdOx1 nCov-19 vaccination, vaccine-induced thrombotic thrombocytopenia (VITT)

## Abstract

**Background:**

Vaccine-induced thrombotic thrombocytopenia (VITT) post SARS-CoV-2 vaccination is characterized by thrombocytopenia and severe thrombosis. Platelet function during patient recovery in the medium-/long-term has not been investigated fully. Here, we undertook a 3-month study, assessing the recovery of a VITT patient and assessing platelet morphology, granule content and dense-granule release at two distinct time points during recovery.

**Case Presentation:**

A 61 year-old female was admitted to hospital 15 days post ChAdOx1 nCov-19 vaccination. Hematological parameters and peripheral blood smears were monitored over 3 months. Platelet morphology and granule populations were assessed using transmission electron microscopy (TEM) at two distinct time points during recovery, as was agonist-induced platelet dense-granule release. Upon admission, the patient had reduced platelet counts, increased D-dimer and high anti-PF4 antibodies with multiple sites of cerebral venous sinus thrombosis (CVST). Peripheral blood smears revealed the presence of large, hypergranular platelets. Following treatment, hematological parameters returned to normal ranges over the study period. Anti-PF4 antibodies remained persistently high up to 90 days post-admission. Two days after admission, VITT platelets contained more granules per-platelet when compared to day 72 and healthy platelets. Additionally, maximal ATP release (marker of dense-granule release) was increased on day 2 compared to day 72 and healthy control platelets.

**Conclusion:**

This study highlights a previously unreported observation of platelet hypergranularity in VITT which may contribute to the thrombotic risk associated with VITT. Optimal approaches to monitoring recovery from VITT over time remains to be determined but our findings may help inform therapeutic decisions relating to anticoagulation treatment in this novel pathology.

## Introduction

Severe acute respiratory syndrome coronavirus 2 (SARS-CoV-2) which causes coronavirus disease 2019 (COVID-19) has caused severe morbidity and mortality globally since the beginning of the pandemic. The most important method of countering the spread of SARS-CoV-2 has been the rapid development and rollout of vaccines against the virus. Currently in Europe, there are four vaccines approved for use by the European Medicines Agency: two messenger RNA vaccines which encode the SARS-CoV-2 spike protein antigen—BNT162b2 (Comirnaty; Pfizer–BioNTech) and mRNA-1273 (Spikevax; Moderna); and two adenoviral vector vaccines encoding the spike protein of SARS-CoV-2—ChAdOx1 nCov-19 (Covishield; Oxford/AstraZeneca) and Ad26.COV2.S (Janssen COVID-19 Vaccine; Johnson & Johnson/Janssen) (1). In March 2021, reports began to emerge of rare adverse clotting events with thrombocytopenia in recipients of the ChAdOx1 nCov-19 (Oxford/AstraZeneca) vaccine, which utilizes a recombinant chimpanzee adenoviral vector encoding the SARS-CoV-2 spike protein (2).

Work from groups in Europe reported three independent cohorts of previously healthy patients who were admitted within 3 weeks of ChAdOx1 nCov-19 vaccination with an unusual presentation of thrombocytopenia and cerebral venous sinus thrombosis (CVST) (1, 3, 4). These groups independently reported patients possessed circulating antibodies against platelet factor 4 (PF4) using anti-PF4-heparin immunoglobulin G (IgG) enzyme-linked immunosorbent assays (ELISA). This condition, vaccine-induced thrombotic thrombocytopenia (VITT), shares similar pathophysiology to heparin-induced thrombocytopenia (HIT) (5), albeit mediated through heparin-independent mechanisms. Due to similarities between VITT and HIT, professional societies have recommended the use of non-heparin anticoagulants, to avoid exacerbation of the condition (6, 7). It is believed that thrombosis in VITT, as in HIT, is caused by PF4/antibody complexes (formed following vaccination) which then bind the platelet membrane receptor FcγRIIA (CD32a), a low affinity Fc receptor, inducing platelet activation (8, 9). Recently, there have been reports of prolonged, persistent anti-PF4 antibodies in VITT patients, but it is believed pathogenic (platelet-activating) antibodies decrease over time (10) and patients do not experience a relapse of the condition (11). To date, the majority of VITT cases have been reported in a minority of recipients of ChAdOx1 nCov-19 or Ad26.COV2.S (Janssen) adenoviral vector vaccines (12). It should also be noted that thrombocytopenia without thrombosis (secondary immune thrombocytopenia) has been reported in small numbers of recipients of the BNT162b2 (Pfizer-BioNTech) and mRNA-1273 (Moderna) mRNA vaccines (13).

Considering the central role platelets play in thrombosis and the VITT clotting pathogenesis (1), there is a dearth of data on the presentation of platelet morphology in VITT patients either upon admission or in their long-term recovery. We, therefore, assessed platelet morphology, granule content and dense-granule release at two distinct time points following admission of a VITT patient to hospital. We also simultaneously tracked the patient's clinical parameters over the same 3-month period, assessing platelet count, D-dimer and anti-PF4 antibody levels to monitor recovery. In this report, day 0 refers to the day of (patient) admission to hospital; all subsequent days listed in this report refer to days post admission to hospital.

## Case Report

A 61-year-old female presented 15 days post ChAdOx1 nCov-19 vaccination (first dose) with a 3-day history of mild headaches (frontal, bilateral radiation) for short periods (12–14 days inclusive) post vaccination. On day 15 a severe headache resulted in nausea and vomiting, following which the patient presented to hospital. Blood tests showed moderate thrombocytopenia (68 × 10^9^/L) and markedly elevated D-dimer levels (17.27 mg/L). C-reactive protein (CRP) levels were increased (20 mg/L), mean platelet volume (MPV) was at the upper limit of the normal range (12 fL) and mild hypoalbuminemia was also observed (33 g/L). Activated partial thromboplastin time (aPTT), prothrombin time (PT) and fibrinogen were all within normal ranges (Table 1). The patient was strongly positive (*OD* = 2.504) for anti-PF4 antibodies by ELISA and heparin-induced platelet aggregation assay (HIPAA) was also positive. Computed tomography venogram showed extensive burden of thrombus involving the right internal jugular vein, right transverse and sigmoid sinuses and the superior sagittal sinus. There was no evidence of raised intracranial pressure or intracranial hemorrhage. Based on these data and published diagnostic guidelines on VITT (13), other immune thrombocytopenic conditions were excluded and a diagnosis of VITT was made.

**Table 1:** Clinical characteristics of VITT patient on admission.

**Characteristic**	
Age (years)	61
Gender	Female
Pre-existing conditions	None
Medication on admission	None
Time from vaccination to admission (days)	15
Symptoms	Headaches, nausea, vomiting
Location of thrombosis	Superior sagittal, right transverse, right sigmoid sinuses; left transverse sinus to lesser extent
Platelets (10^9^/L) (150–400)	68
D-dimer (mg/L) (0.0–0.5)	17.27
aPTT (s) (25.0–36.5)	30.7
PT (s) (10.4–13.0)	12.5
Fibrinogen (g/L) (1.5–4.0)	1.7
CRP (mg/L) (<7)	20
Albumin (g/L) (35.0–40.0)	33.0
MPV (fL) (7.5–12.0)	12.0

*Reference ranges of platelet count, D-dimer, aPTT, PT, fibrinogen, CRP, albumin, and MPV are shown in parentheses. aPTT, activated partial thromboplastin time; PT, prothrombin time; CRP, C-reactive protein; MPV, mean platelet volume*.

**Figure 1:**
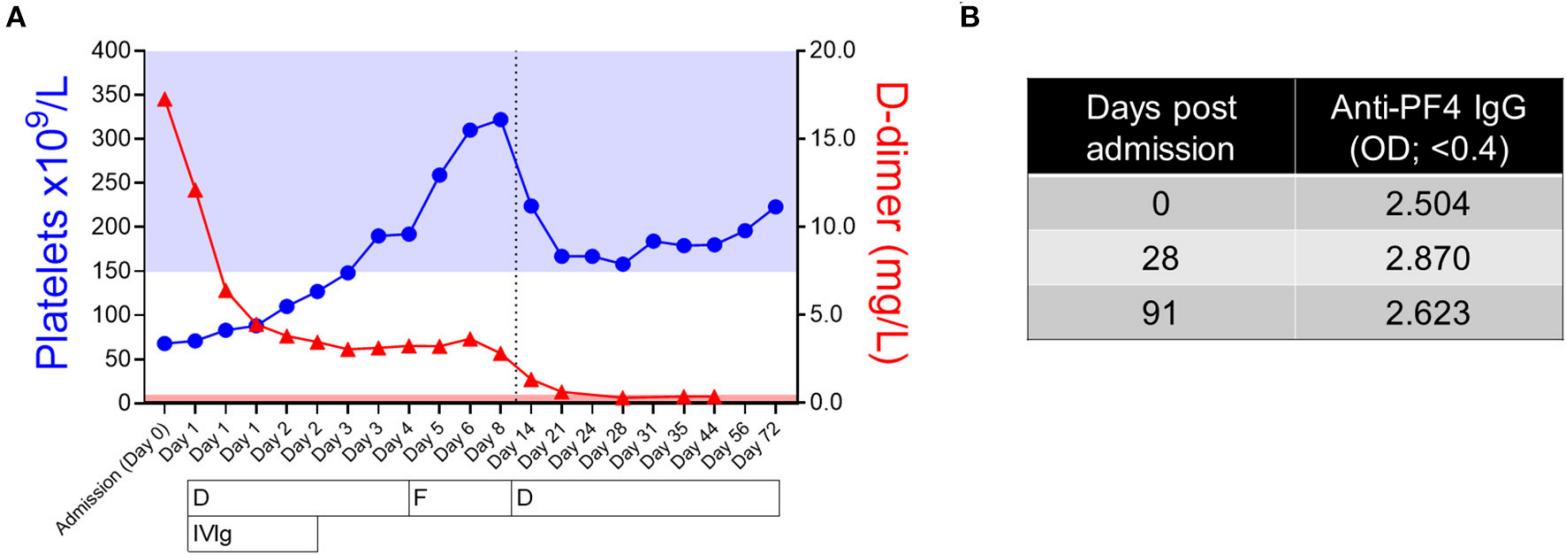
Clinical timelines of patient's platelet count, D-dimer levels, anticoagulation treatment and anti-Platelet Factor 4 (PF4) antibody levels. **(A)** Patient platelet count (platelets × 10^9^/L; blue closed circles) and D-dimer (mg/L; red closed triangles) are plotted over time from day of admission (day 0) to day 72. Note presence of three separate values for day 1 and two values each for days 2 and 3, respectively; indicates multiple values taken over the course of 1 day. Reference ranges of platelet count (150–400 × 10^9^/L) and D-dimer (0.0–0.5 mg/L) are indicated by the blue (platelet count) and red (D-dimer) shaded areas. The vertical dashed line indicates the time of patient discharge (Day 9): values after this line were collected at outpatient appointments. Missing values for D-dimer: days 24, 31, 56, and 72. Timing of anticoagulation and intravenous immune-globulin (IVIg) are indicated under the graph. D; dabigatran (150 mg; twice daily), F; fondaparinux (7.5 mg; once daily, subcutaneous). **(B)** Patient anti-PF4 antibody levels (measured by Immucor PF4 IgG ELISA) from three time points over 13 weeks [day of admission (day 0), day 28, and day 91]. Results of OD > 0.4 are considered positive for circulating anti-PF4 antibodies.

The patient was treated with intravenous immunoglobulin (IVIg; 1 g/kg, 2 days) and dabigatran [150 mg: twice daily initially (to facilitate reversibility during thrombocytopenic phase if required)]. Anticoagulation was changed to fondaparinux (7.5 mg: once daily subcutaneous for 5 days) following platelet count recovery before reverting to dabigatran (150 mg; twice daily) (Figure 1A). After 3 days, platelet count returned to normal range (190 × 10^9^/L), while D-dimer reduced significantly but remained elevated (3.12 mg/L) (Figure 1A), in keeping with other reports (11). The patient was discharged on day 9 with symptoms resolved and with continued dabigatran (150 mg: twice daily). The patient agreed and actively cooperated with the above treatment both during and post hospitalization. Follow-up assessments showed sustained platelet count and D-dimer within normal ranges (Figure 1A). Interestingly, anti-PF4 antibodies remained persistently high up to 90 days post admission (Figure 1B) as observed by others (10, 11).

**Figure 2:**
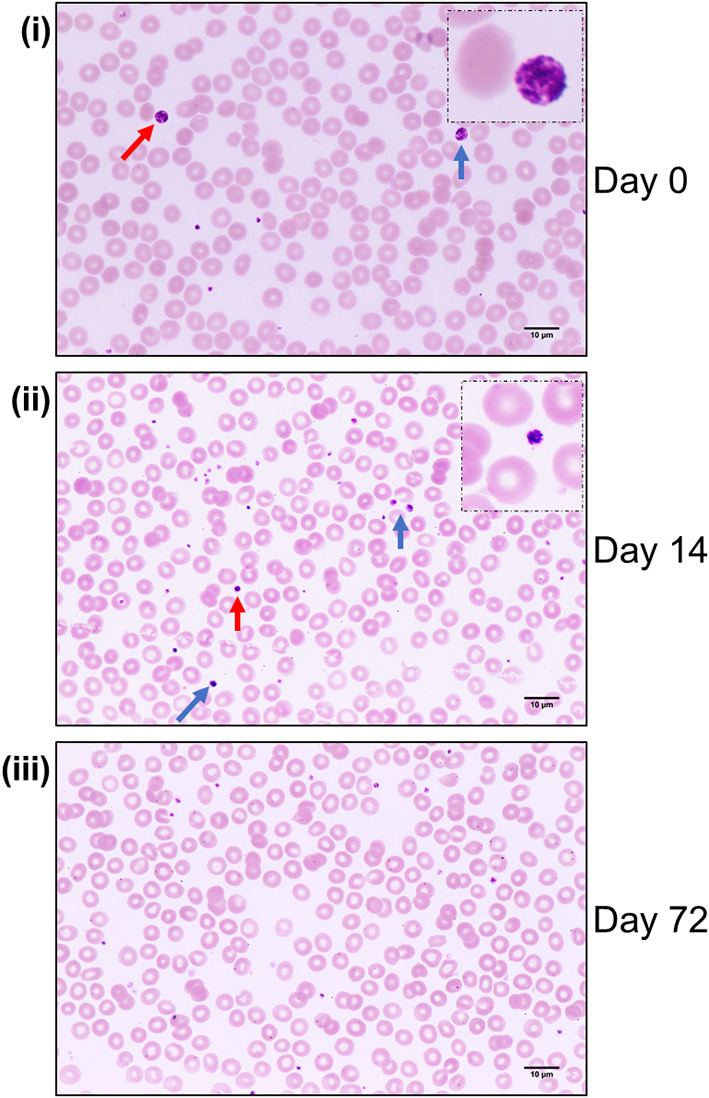
Peripheral blood smears in VITT reveal populations of large, hypergranular platelets that decrease over time. Peripheral blood smears performed on day of admission [day 0 **(i)**] and days 14 **(ii)** and 72 **(iii)** post admission. Colored arrows denote presence of large, hypergranular platelets **(i)** or hypergranular platelets **(ii)**. Dash-frame inserts in C **(i)** and **(ii)** are enlarged areas of platelets highlighted by red arrows in the corresponding main image. Images were captured using a Nikon Eclipse 80i light microscope with Nikon Plan Apo VC 100 × /1.40 0.17 oil immersion lens, using a Canon EOS 600D camera. Stained with Wright Giemsa. Magnification: 100 ×.

Peripheral blood smears performed on day of admission (day 0) showed evidence of thrombocytopenia and revealed the presence of distinctive large, hypergranular platelets (Figure 2i), corresponding with the previously observed MPV at the upper limit of normal range on admission (Table 1). These platelets possessed an average diameter of 5.4 ± 0.97 μm and an average incidence rate of ~10.5 × 10^9^/L. Subsequent blood smears performed on day 14 showed no evidence of thrombocytopenia or large platelets but populations of highly granular platelets (average diameter 3.6 ± 0.92 μm) continued to persist (approximate average incidence rate 1.9 × 10^9^/L) (Figure 2ii). By day 72, blood smears revealed a normal platelet morphology (average diameter 3.5 ± 0.74 μm) with no evidence of thrombocytopenia or large, hypergranular platelets present (Figure 2iii). Though schistocytes have been reported in VITT patients (14), we observed no evidence of them in this study.

**Figure 3:**
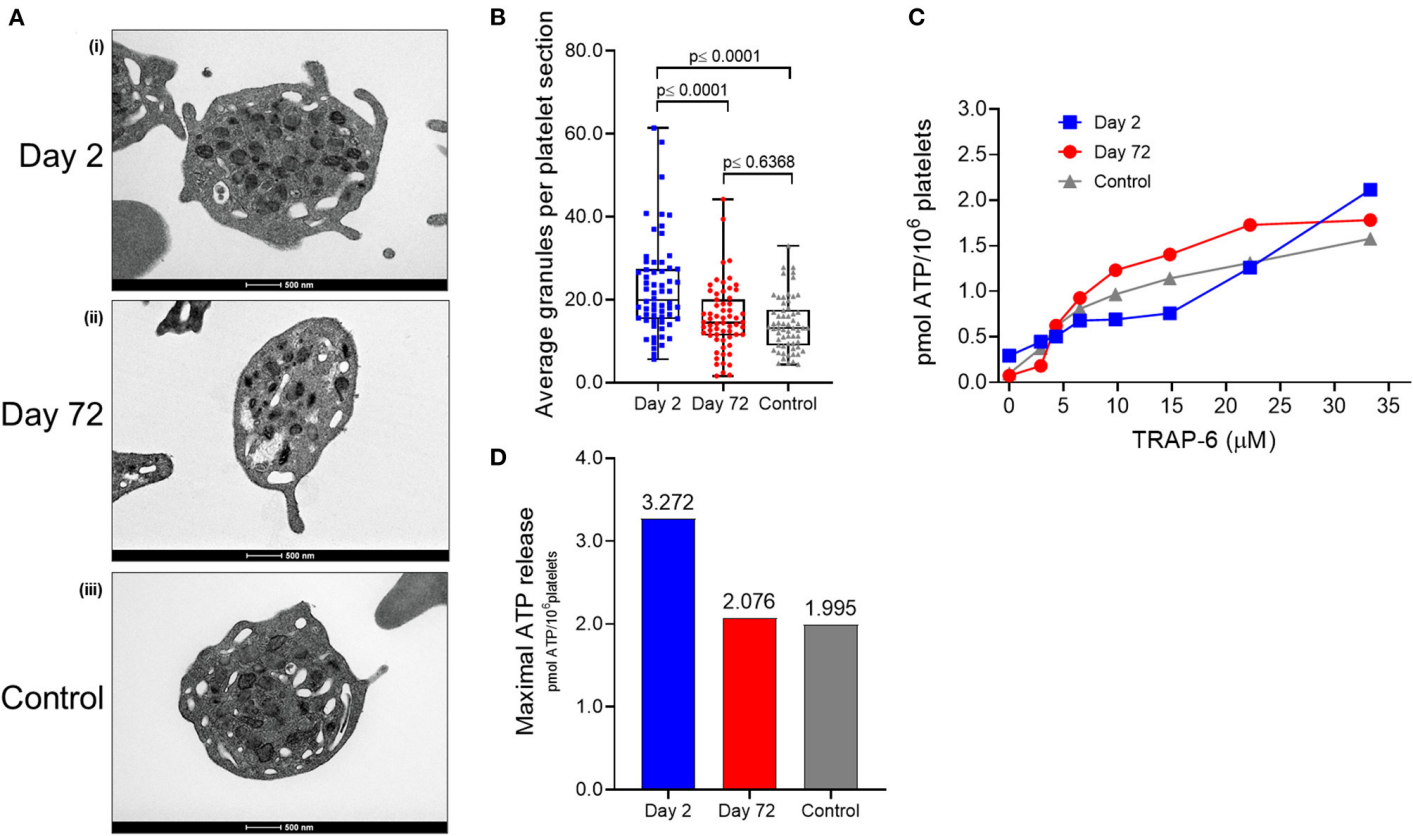
Average platelet section granule numbers and dense-granule release in VITT. **(A)** Representative transmission electron microscopy (TEM) images of ultra-thin sections of platelets from VITT patient on day 2 (i) and day 72 (ii) post admission, and healthy control (iii). Scale bars at bottom of images represent 500 nm. Platelets were stained with 2% uranyl acetate and 3% lead citrate. Magnification: 20,500 ×. Images are representative for average platelet-section granule numbers per cohort as presented in **(B)**. **(B)** The number of all granule types (α- and dense-granules, and lysosomes) per platelet section were semi-quantified in one cross-sectional plane by five individuals and averaged. Counting was blinded to avoid bias and structures identified as granules were typical of α- and dense-granules, and lysosomes as previously reported (15). Boxplots represent the data median (line inside the box; day 2-19.5; day 72-14.5; healthy control-13.5) and the interquartile range [IQR; outline of the box; day 2 (15, 26.75); day 72 (11.4, 20); healthy control (9, 18)] together with data maximum (day 2-61.4; day 72-44.2; healthy control-27.08) and data minimum (day 2-5.6; day 72-1.9; healthy control-4.4) (whiskers) and averaged individual observations for each platelet section. Differences in granule count were assessed using a One-way ANOVA with an FDR of 5%. Further pair-wise comparisons were performed using a Tukey honestly significant difference (HSD) test based on a 95% family-wise confidence level. Significance (*p*) values between cohorts are denoted above the boxplots. **(C)** Platelet dense-granule release was measured in duplicate in day 2 and 72 VITT, and healthy control platelets. ATP secretion (marker of dense-granule secretion) was measured using a luminescence-based assay. Data was expressed as the amount of ATP secreted, in luminescence arbitrary units, and converted to pmol ATP released per 10^6^ platelets by comparison with the luminescence recorded from an ATP standard (0.4 mM) and plotted as a dose response curve. Day 2 platelets did not achieve agonist saturation as can be observed from continued increases in ATP release at higher doses, compared to day 72 and healthy control platelets. **(D)** Maximal ATP release (pmol ATP/10^6^ platelets) in response to high dose TRAP-6 (33.3 μM). Experimental procedures of all data presented in this Figure can be found in the Supplementary Material.

Based on the platelet presentation in day 0 peripheral blood smears, platelet transmission electron microscopy (TEM) was performed on VITT platelets at two distinct points in the patient's recovery, 2 days, and 72 days post admission to hospital. TEM on platelets from a gender-not-age-matched healthy control were also assessed to investigate platelet morphology and comparatively quantify platelet granule number in VITT (see Supplementary Material for experimental procedures). Washed platelets (see Supplementary Material) were fixed, and TEM images of 62 randomly selected individual platelet sections from the three separate cohorts (VITT day 2, VITT day 72, and healthy control) were used for morphology characterization and granule quantification (Figure 3A). VITT platelet sections on day 2 (Figure 3Ai) appeared, on average, to contain a greater number of granules compared to VITT platelet sections on day 72 and healthy control platelet sections (Figures 3A**ii,iii**). This was reflective of the MPV for the 3 cohorts at the time of blood donation: 12.2 fL (day 2); 11.2 fL (day 72); 9.2 fL (healthy control). The average granule count (α- and dense-granules and lysosomes) per individual platelet section was quantified for all 3 cohorts. We found VITT day 2 platelet sections contained significantly more granules when compared to both VITT day 72 and healthy control platelet sections (Figure 3B), akin to the increased presence of hypergranular platelets in the day 2 blood smears. The mean granule count for each cohort was: day 2—22.052 [Interquartile range (IQR) (15, 26.75)]; day 72—15.541 [IQR (11.4, 20)]; healthy control—14.419 [IQR (9, 18)] (Figures 3A,B). No statistically significant difference was found between VITT day 72 and healthy control platelet sections (Figure 3B).

Given the observed hypergranular appearance of the VITT platelets (Figures 2, 3A), we next assessed platelet dense-granule release using an agonist-induced platelet adenosine triphosphate (ATP) secretion assay (see Supplementary Material for experimental procedure), measuring ATP secretion in response to a range of thrombin receptor activator peptide-6 (TRAP-6) doses (Figures 3C,D). Interestingly, we observed that VITT day 2 platelets released more ATP in response to high dose TRAP-6 compared to VITT day 72 and healthy control platelets (Figure 3D). Intriguingly, VITT day 2 platelets did not achieve saturation of dense-granule release in response to increasing doses of TRAP-6 when compared to day 72 and healthy control platelets (Figure 3C).

## Discussion

Despite the success of COVID-19 vaccination programs across the world, VITT has emerged as a new, albeit rare, clinical challenge, associated with severe morbidity and mortality. The primary VITT-inducing ChAdOx1 nCov-19 (AstraZeneca) or Ad26.COV2.S (Janssen) vaccines employ replication-incompetent adenoviral vectors and it is theorized that an anionic component, of the vaccine or produced by cells at vaccination sites, induces anti-PF4 antibody generation which in turn induce platelet activation via the FcγRIIA receptor (5). However, at the time of writing, the specific VITT-inducing vaccine component remains unknown (12).

Our work highlights a previously unreported observation of platelet hypergranularity in the early stages of VITT with increased capacity for secretion and, possibly, platelet-hyperactivity contributing to vaccine-induced thrombosis. The hypergranular platelets may have been immature platelets, known to be prothrombotic and hyper-reactive with high dense-granules content (16) and are increased in other immune thrombocytopenic conditions such as immune thrombocytopenic purpura and thrombotic thrombocytopenic purpura (17), contributing to thrombotic events. The increased presence of immature platelets is most likely caused by platelet overproduction, secondary to platelet consumption or hypoproduction (17). The reduction in hypergranular/immature platelet populations over time in our patient was most likely caused by the resolution of platelet count to normal range, contributing to the decrease in platelet granule counts and dense-granule secretion (ATP release) we observed between VITT platelets from days 2 and 72, feasibly reducing our patient's thrombotic risk.

The extent of thrombotic events in VITT patients has been striking in studies to date. CVST continues to be the predominant thrombotic event but others such as pulmonary embolism and deep-vein thrombosis have been found (18). Patient presentation of low platelet count (≤ 30 ×10^9^/L) and elevated D-dimer, coupled with increased coagulation activation and CVST, are associated with increased mortality in VITT patients as reported in a recent prospective cohort study from the United Kingdom (18). It is thought that VITT-induced platelet activation leads to increased release of platelet large extracellular vesicles (EVs; microparticles) which are known to contain tissue factor, which is believed to be the main driver of CVST in VITT (12, 19).

Since this pathology was first identified, treatment of VITT patients has been primarily based on treatments employed for HIT: use of non-heparin anticoagulants such as direct thrombin inhibitors (DTIs; argatroban) and Factor Xa inhibitors (apixaban, fondaparinux) and avoidance of platelet transfusions and heparin administration (5, 12). Early IVIg administration has also become an important component of initial acute management of VITT, a treatment strategy which has also been utilized in cases of complicated HIT including atypical and autoimmune HIT (5, 18). VITT cases that are unresponsive to the above-described treatments (refractory VITT) have also been reported and therapeutic plasma exchange has been proposed as a treatment for cases of refractory VITT (20). Bruton's tyrosine kinase (Btk) inhibitors have also been proposed as a possible treatment for VITT as they target various processes downstream of FcγRIIA such as platelet aggregation and dense granule secretion (21). Dabigatran (univalent DTI) and fondaparinux (indirect Factor Xa inhibitor) were used in the treatment of our patient and their effects, coupled with IVIg, were immediate with platelet count and D-dimer levels resolving drastically over the first several days post admission. Dabigatran was administered rather than argatroban to deliver robust anticoagulation—unhampered by argatroban monitoring challenges (22)—and due to dabigatran's reversibility while platelet count was reduced and bleeding risk high.

Lastly, akin to a recent study (10), we observed prolonged anti-PF4 antibodies in this patient despite IVIg and anticoagulation treatment. Schönborn et al. found anti-PF4 antibodies can persist in VITT patients but pathogenic antibodies decrease over time (10). The same study also suggested pathogenic anti-PF4 antibodies may persist for >12 weeks in a small subgroup of patients, which necessitates further study to clarify if prolonged anticoagulation may be required. Thaler et al. also reported similar observations, with anti-PF4 antibodies decreasing but at a very slow rate over several weeks (11). Due to these persistent anti-PF4 antibodies, in the United Kingdom it has been proposed that antibody levels be monitored weekly for the first 4 weeks and monthly for 6 months thereafter (12). Whether long-term anticoagulation is also necessary is still to be fully elucidated (11, 12).

Limitations of this report include the lack of an appropriately age-and-vaccine status matched control for comparative platelet analyses. A more appropriate control would have been a healthy, age-matched control who had received the first dose of the ChAdOx1 nCov-19 vaccine without experiencing adverse reactions/events. However, due to public health restrictions that were in place during the period of this case, recruitment of more appropriate healthy controls was not possible. Another limitation is the assessment of platelet granules. Advanced electron microscopy techniques such as 3-dimensional ultrastructural analysis allow for detailed analysis of granule populations in platelets (23), however, this technology was not available to us. Our analysis was of platelets in a single sectional plane which does not allow for a global assessment of granule populations in a platelet but rather per platelet section. While our data are reflective of MPV values and of hypergranular platelet populations seen in peripheral blood smears, global platelet granule population assessment would give a more accurate picture of platelet granule populations in VITT.

In conclusion, we have presented the first report of hypergranular platelets (possibly prothrombotic immature platelets) in VITT which may contribute to the clotting pathogenesis observed in VITT. We have learned and continue to learn a significant amount about this new pathology since the first reports emerged in March 2021. Important next steps in optimizing the management of this novel condition will include defining the optimal duration of anticoagulant treatment and determining the long-term outcomes among affected patients. The optimal approach to monitoring of anti-PF4 antibody levels, and other laboratory parameters, over time also remains to be determined but may help inform therapeutic decisions.

## Patient Perspective

The patient said that after receiving her diagnosis of VITT, she was unable to fully grasp what had occurred, and did not fully understand the gravity of the situation. The patient attributes this to her clouded consciousness, however, she states that 4 days post admission this began to subside. The patient feels incredibly fortunate that hospital staff were so proactive in her care and to have been treated in such a specialized hospital. Before she realized that she was so sick the patient said she was already getting better, responded well to treatment, and said that she “came from a very sick place to improving within a few days.” It was not until ~4 months after her diagnosis that the patient recalls fully understanding the magnitude of this complication. Upon reflection the patient describes her diagnosis as being unfortunate, that she fell ill while trying to do the right thing—however, she states that is something she does not want to dwell on. The patient describes her experience with hospital staff and her treating physicians as being very positive. She wishes to thank hospital staff during her stay in hospital for being so proactive and double-checking her scans. The patient believes that “timing is everything.” Regarding the care she received, the patient said she is “amazed by the support [she] received from day one.” She recalls everyone involved in her care as being readily available and was grateful to have access to a support nurse whom she could contact via phone or email with any query. Through this experience, the patient became aware of several “services [she] had never availed of, or even knew existed, [and is] happy to know they are there. Makes it all easier.” We are grateful to the patient for sharing her experience with us and consenting to it being included in this report.

## Data Availability Statement

The original contributions presented in the study are included in the article/Supplementary Material, further inquiries can be directed to the corresponding author/s.

## Ethics Statement

The studies involving human participants were reviewed and approved by Institutional Review Board of the Mater Misericordiae University Hospital. The patients/participants provided their written informed consent to participate in this study. Written informed consent was obtained from the individual(s) for the publication of any potentially identifiable images or data included in this article.

## Author Contributions

SPC, AL, PS, PM, FNÁ, and BK designed the research. SPC, AL, KS, OE, SK, CH, ÁL, FNÁ, and BK collected clinical data. FNÁ was responsible for patient care and treatment. SPC, KS, and SK performed peripheral blood smear microscopy. SPC, TO'N, and NS performed platelet transmission electron microscopy and morphology assessment. SPC, AL, OE, CM, SR, and AT performed platelet section granule analysis. SC and NM performed the ATP secretion assay. SPC, AL, SC, LW, and NM analyzed the data. SPC, AL, PM, FNÁ, and BK wrote the manuscript. All authors reviewed and approved the final manuscript.

## Funding

This research is part of the COVID COCOON study which was funded by a COVID-19 Rapid Response Grant (20/COV/0157) from Science Foundation Ireland awarded to BK. The funders had no role in study design, data collection and analysis, decision to publish, or preparation of the manuscript.

## Conflict of Interest

SPC is the Sanofi S.A. Newman Fellow in Haematology. Sanofi S.A. had no input into study design, data collection and analysis, decision to publish, or preparation of the manuscript. The remaining authors declare that the research was conducted in the absence of any commercial or financial relationships that could be construed as a potential conflict of interest.

## Publisher's Note

All claims expressed in this article are solely those of the authors and do not necessarily represent those of their affiliated organizations, or those of the publisher, the editors and the reviewers. Any product that may be evaluated in this article, or claim that may be made by its manufacturer, is not guaranteed or endorsed by the publisher.
