# Supplementary material for: Case Report: Hypergranular Platelets in Vaccine-Induced Thrombotic Thrombocytopenia After ChAdOx1 nCov-19 Vaccination
Source: Front Cardiovasc Med. 2022 Feb 9;9:824601. doi: 10.3389/fcvm.2022.824601 (PMC8865139; doi:10.3389/fcvm.2022.824601)
Supplement: Supplementary file 1 [file Data_Sheet_1.docx]

**SUPPLEMENTARY MATERIAL:**

**Supplementary Experimental Procedures:**

**Blood collection and platelet preparation:**

Whole blood was collected from the patient and healthy control, with informed consent under the Declaration of Helsinki. Ethical approval was granted by the Institutional Review Board of the Mater Misericordiae University Hospital (1/378/1821). Washed platelets were prepared as previously described (1). In brief, blood was drawn via venepuncture with a 21-gauge needle into 10 mL acid citrate dextrose-A (ACD-A) vacutainers and centrifuged (200 xg, no brake) for 15 minutes at room temperature (RT). Platelet rich plasma (PRP) was isolated, pH adjusted to 6.5 with ACD-A, and supplemented with 1 μM PGE_1_. Erythrocytic contamination was removed from PRP by centrifugation (150 xg, 7 min, RT). Platelets were isolated from plasma by centrifugation (600 xg, 10 min, RT). Isolated platelets were washed using a modified Tyrode’s buffer (130 mM NaCl, 9 mM NaHCO3, 10 mM Tris-HCl, 10 mM Trisodium citrate, 3 mM KCl, 0.81 mM KH2PO4, 9 mM MgCl2 x 6H2O; pH 7.4 with ACD-A) followed by centrifugation (600 xg, 10 min at RT). Platelets were again resuspended in modified Tyrode’s buffer and incubated at 37^o^C.

**Platelet Transmission Electron Microscopy (TEM):**

Washed platelets (described above) were fixed in 2.5% glutaraldehyde in Sørensen’s phosphate buffer (SPB) at RT. Samples were briefly rinsed with SPB, post-fixed in 1% osmium tetroxide in SPB for 1 hour and rinsed again with SPB. Subsequently, samples were dehydrated in a graded ethanol series (30%, 50%, 70%), stained with saturated uranyl acetate in 70% ethanol and dehydration was completed with 90% and 100% ethanol. After dehydration, samples were immersed in acetone, then transferred to an Acetone/Epon resin mixture (1:1 vol/vol) for 1 h. To complete resin infiltration, samples were placed in pure resin for 2 hours at + 37 °C. Final polymerization was carried out at + 60 °C for 24 h. Ultrathin (80 nm) sections were obtained with a diamond knife using Leica EM UC7 ultramicrotome. Sections were collected on 200 mesh thin bar copper grids, stained with 2% uranyl acetate (20 min) and 3% lead citrate (5 min). Sections were examined by TEM (Tecnai G2 12 BioTWIN) 120kV accelerating voltage.

**Platelet Granule Quantification:**

62 platelet sections per-condition were assessed for platelet granule content using transmission electron micrographs. Total number of all platelet granule types (α-/dense-granules, and lysosomes) were semi-quantified in one cross-sectional plane per-platelet section, by five individuals and then averaged. A blinded count was performed to avoid bias and structures identified as granules were typical of α-/dense-granules, and lysosomes as previously described (2). Granule counting was performed using the cell counter plugin in Image J (3).

**ATP secretion assay:**

ATP secretion was measured using a luminescence-based assay, as previously described (4, 5). Agonists and buffer (10 μl) were dispensed in duplicate into 96-well plates, together with 70μl of platelet-rich-plasma (isolation described above) and incubated for 3 minutes at 37 °C, with fast orbital shaking. Doses of thrombin receptor activating peptide-6 (TRAP-6; T1573-5MG, Sigma-Aldrich) used to induce platelet ATP secretion were: 2.9 μM, 4.3 μM, 6.5 μM, 9.8 μM, 14.8 μM, 22.2 μM, 33.3 μM. Following incubation, 5μl of ATP detecting reagent (chronolume; Labmedics, UK) was dispensed into each well and luminescence was measured immediately using a Perkin Elmer Platelet 1420 96-well plate reader. Data was expressed as the amount of ATP secreted, in luminescence arbitrary units (AU), and converted to pmol ATP released per 10^6^ platelets by comparison with the luminescence recorded from an ATP standard (0.4 mM).

**References**

1. Parsons MEM, Szklanna PB, Guerrero JA, Wynne K, Dervin F, O'Connell K, et al. Platelet Releasate Proteome Profiling Reveals a Core Set of Proteins with Low Variance between Healthy Adults. PROTEOMICS. 2018;18(15):1800219.

2. Neumüller J, Ellinger A, Wagner T. Transmission electron microscopy of platelets from apheresis and buffy-coat-derived platelet concentrates. The Transmission Electron Microscope: Intechopen London: Intechopen. 2015:255-84.

3. O'Brien J, Hayder H, Peng C. Automated Quantification and Analysis of Cell Counting Procedures Using ImageJ Plugins. J Vis Exp [Internet]. 2016 2016/11//; (117).

4. Mitrugno A, Williams D, Kerrigan SW, Moran N. A novel and essential role for FcgammaRIIa in cancer cell-induced platelet activation. Blood. 2014;123(2):249-60.

5. Comer SP, Cullivan S, Szklanna PB, Weiss L, Cullen S, Kelliher S, et al. COVID-19 induces a hyperactive phenotype in circulating platelets. PLOS Biology. 2021;19(2):e3001109.
